# ^68^Ga-DOTA-Siglec-9 PET/CT imaging of peri-implant tissue responses and staphylococcal infections

**DOI:** 10.1186/s13550-014-0045-3

**Published:** 2014-08-08

**Authors:** Helena Ahtinen, Julia Kulkova, Laura Lindholm, Erkki Eerola, Antti J Hakanen, Niko Moritz, Mirva Söderström, Tiina Saanijoki, Sirpa Jalkanen, Anne Roivainen, Hannu T Aro

**Affiliations:** 1 Turku PET Centre, Turku University Hospital, University of Turku, Turku FI-20521, Finland; 2 Orthopaedic Research Unit, Department of Orthopaedic Surgery and Traumatology, Turku University Hospital, University of Turku, Turku FI-20521, Finland; 3 Department of Medical Microbiology and Immunology, University of Turku, Turku FI-20521, Finland; 4 Antimicrobial Resistance Unit, National Institute for Health and Welfare, Turku FI-20521, Finland; 5 Turku Clinical Biomaterials Centre, Institute of Dentistry, University of Turku, Turku FI-20521, Finland; 6 Department of Pathology, Turku University Hospital, University of Turku, Turku FI-20521, Finland; 7 MediCity Research Laboratory, University of Turku, Turku FI-20521, Finland; 8 Turku Center for Disease Modeling, University of Turku, Turku FI-20521, Finland

**Keywords:** 68Ga-DOTA-Siglec-9, Implant infection, Osteomyelitis, PET, S. aureus, S. epidermidis, VAP-1

## Abstract

**Background:**

*Staphylococcus epidermidis* (*S. epidermidis*) has emerged as one of the leading pathogens of biomaterial-related infections. Vascular adhesion protein-1 (VAP-1) is an inflammation-inducible endothelial molecule controlling extravasation of leukocytes. Sialic acid-binding immunoglobulin-like lectin 9 (Siglec-9) is a leukocyte ligand of VAP-1. We hypothesized that ^68^Ga-labeled 1,4,7,10-tetraazacyclododecane-1,4,7,10-tetraacetic acid-conjugated Siglec-9 motif containing peptide (^68^Ga-DOTA-Siglec-9) could detect inflammatory response due to *S. epidermidis* peri-implant infection by positron emission tomography (PET).

**Methods:**

Thirty Sprague-Dawley rats were randomized into three groups. A sterile catheter was implanted into the medullary canal of the left tibia. In groups 1 and 2, the implantation was followed by peri-implant injection of *S. epidermidis* or *Staphylococcus aureus* (*S. aureus*) with adjunct injections of aqueous sodium morrhuate. In group 3, sterile saline was injected instead of bacteria and no aqueous sodium morrhuate was used. At 2 weeks after operation, ^68^Ga-DOTA-Siglec-9 PET coupled with computed tomography (CT) was performed with the measurement of the standardized uptake value (SUV). The presence of the implant-related infection was verified by microbiological analysis, imaging with fluorescence microscope, and histology. The *in vivo* PET results were verified by *ex vivo* measurements by gamma counter.

**Results:**

In group 3, the tibias with implanted sterile catheters showed an increased local uptake of ^68^Ga-DOTA-Siglec-9 compared with the intact contralateral bones (SUV_ratio_ +29.5%). ^68^Ga-DOTA-Siglec-9 PET detected inflammation induced by *S. epidermidis* and *S. aureus* catheter-related bone infections (SUV_ratio_ +58.1% and +41.7%, respectively). The tracer uptake was significantly higher in the *S. epidermidis* group than in group 3 without bacterial inoculation, but the difference between *S. epidermidis* and *S. aureus* groups was not statistically significant. The difference between the *S. aureus* group and group 3 was neither statistically significant.

**Conclusion:**

PET/CT imaging with novel ^68^Ga-DOTA-Siglec-9 tracer was able to detect inflammatory tissue response induced by catheter implantation and staphylococcal infections.

## Background

Coagulase-negative staphylococci, including *Staphylococcus epidermidis*, have emerged as the leading pathogen of nosocomial implant-related infections, including periprosthetic joint infections [1] and intravascular catheter-related bloodstream infections [2]. Slime-producing *S. epidermidis* strains exhibit robust attachment to the plastic devices and metallic implant surfaces followed by slow proliferation and low metabolic activity within the biofilm [3]-[5]. By nature, these infections are frequently clinically indolent and represent diagnostic and treatment challenges [1],[6],[7]. Related to differences in virulence, the outcome of bone implant infections caused by coagulase-negative staphylococci is better than those caused by *Staphylococcus aureus* (*S. aureus*) [8].

The gold standard for the non-invasive imaging of periprosthetic joint infections is the use of white blood cell (WBC) scintigraphy [9],[10]. The American Academy of Orthopaedic Surgeons (AAOS) could give only a weak recommendation for nuclear imaging modalities in the diagnosis of periprosthetic joint infections [11]. The inaccuracy of [^18^Fluorine]-2-fluoro-2-deoxyglucose combined positron-emission tomography/computed tomography (^18^F-FDG PET/CT) imaging relates not only to the high uptake of the tracer both in bacterial infections and aseptic inflammatory processes, such as mechanical loosening of prostheses [12], but probably also to the difficulties in detection of indolent low-grade *S. epidermidis* infections.

Confirming the clinical experience with ^18^F-FDG-PET imaging of chronic osteomyelitis, our experimental studies of rabbit tibia models have shown that ^18^F-FDG-PET imaging is highly effective in detection of *S. aureus* osteomyelitis [13], in evaluation of prevention of *S. aureus* biomaterial infections [14], and in evaluation of treatment response in local therapy of *S. aureus* osteomyelitis [15]. In contrast, our recent experiment confirmed that sub-acute peri-implant *S. epidermidis* infections are characterized by low ^18^F-FDG uptake in the rabbit osteomyelitis model [16]. The result demonstrated the need of better PET tracers for diagnosing *S. epidermidis* infections.

Leukocyte migration is an important step in several types of acute and chronic inflammation as well as autoimmune diseases. Vascular adhesion protein-1 (VAP-1) is an inflammation inducible 170-kDa endothelial sialoglycoprotein mediating interaction between leukocyte and endothelium [17],[18]. VAP-1 is stored in intracellular granules within endothelial cells. However, upon inflammation, it is rapidly translocated to the endothelial cell surface. Besides being an adhesion molecule, VAP-1 is also a semicarbazide-sensitive amine oxidase (SSAO) enzyme, which catalyzes oxidative deamination of primary amines resulting in aldehyde formation and releasing of hydrogen peroxide [19]. The end products are highly potent inflammatory mediators. Therefore, VAP-1 is both an optimal candidate for anti-inflammatory therapy and a potential target for imaging of inflammation.

Sialic acid-binding immunoglobulin-like lectins (Siglecs) are usually involved during inflammatory and immune responses in subset of leukocytes [20]. We have recently discovered that Sialic acid-binding immunoglobulin-like lectin 9 (Siglec-9) is a leukocyte ligand of VAP-1 and a Gallium-68-labelled 1,4,7,10-tetraazacyclododecane-1,4,7,10-tetraacetic acid (DOTA)-conjugated Siglec-9 motif peptide (^68^Ga-DOTA-Siglec-9) can be used for PET imaging of inflammation and cancer [21].

The current study was delineating the efficacy of novel ^68^Ga-DOTA-Siglec-9 PET for the detection of inflammatory response due to *S. epidermidis* peri-implant infection. The comparison was made with implant infections caused by *S. aureus* and a sterile implant group.

## Methods

### Experimental design

Thirty adult male rats were randomized into three groups. Each animal underwent surgical implantation of a sterile intravenous catheter into the medullary canal of the left tibia while the right tibia served as the intact intra-animal control. In group 1, the implantation was followed by sequential injections of aqueous sodium morrhuate and biofilm-inducing *S. epidermidis* suspension via the catheter. The animals of group 2 received equal injections of aqueous sodium morrhuate and biofilm-inducing *S. aureus* suspension. In group 3, an equal amount of sterile saline was injected via the sterile catheter. Two weeks after surgery, PET/CT imaging with ^68^Ga-DOTA-Siglec-9 tracer was performed. The *in vivo* PET results were verified by *ex vivo* measurements of both tibias. The presence of inoculated staphylococcal infections and the absence of contamination in the group with sterile catheters were verified by separate microbiological analyses of bone specimens and retrieved catheters. The presence of microbial biofilms on catheters was verified *ex vivo* with fluorescence microscopy. Histological inflammatory reactions were graded using a scoring system.

### Ethical statement

The animal study protocol was approved by the Finnish National Animal Experiment Board, ELLA (Permit # ESAVI/3485/04.10.03/2012). The animal experiments were carried out in the Central Animal Laboratory of the University of Turku. The institutional guidelines and the protocols for the analgesia, anesthesia, and housing of the rats were followed. Before surgery, the rats were acclimated to their new environment and fed a standard laboratory diet. The animals were housed in groups of two with constant room temperature. After surgery, the functional activity of the animals was not restricted. The animals were allowed free weight-bearing after recovery from anesthesia.

### Animals

Thirty adult male Sprague-Dawley rats (obtained from Harlan, the Netherlands), weighing a mean of 425 g (SD 37 g) were used. Five rats served as reserve.

### Bacterial strains and measurement of biofilm production capability

*S. epidermidis* clinical isolate T-54580 and *S. aureus* clinical isolate 52/52A/80 were used. Prior to the *in vivo* experiment, the bacterial strains were tested for their *in vitro* capability to form the biofilm. The strains were cultured overnight at 35°C with agitation on brain-heart infusion broth (BHI; Sigma-Aldrich, co, St. Louis, MO, USA). Thereafter, the bacterial suspension was adjusted to an optical density (OD) at 600 nm to 0.18 in BHI. Static biofilms were constructed according to Merrit et al. [22]. Briefly, the bacterial suspensions were diluted with BHI in a ratio of 1:10, and 200 μL were pipetted into the 96-well flat bottom polystyrene microplates (Nunc A/S, Roskilde, Denmark). BHI without the bacterial suspension was used as a control. Bacteria were incubated at 35°C, in ambient air for 24 h. After incubation, the culture medium was removed and the wells were washed twice with phosphate buffered saline (PBS) to remove planktonic cells. Capability of biofilm formation was analyzed by the crystal violet technique [22]. All tests were performed in triplicate.

### Preparation of bacterial suspension for the in vivo study

*S. epidermidis* and *S. aureus* were cultured overnight on blood agar plates. Thereafter, bacterial suspensions were prepared by adjusting OD at 600 nm to 0.18 (corresponding to Mc Farland 1) in sterile saline. One milliliter of *S. epidermidis* suspension was adjusted to be equal to 3 × 10^8^ colony-forming units (CFU). One milliliter of *S. aureus* suspension was diluted to be equal to 3 × 10^5^ CFU. Bacterial suspensions were stored at 4°C and used as an inoculum at the day of preparation. To evaluate the actual bacterial number in each suspension, the series of tenfold dilutions were prepared and 100 μL from each dilution was plated on blood agar plates to calculate colony-forming units per milliliter.

### Animal model

For surgery, the animals were anesthetized by subcutaneous injections of a mixture of ketamine hydrochloride (Ketaminol® vet 50 mg/mL, Intervet International B.V., Boxmeer, the Netherlands) and medetomidine hydrochloride (Cepetor vet 1 mg/mL, CP-Pharma Handelsges. mbH, Burgdorf, Germany). Skin preparation involved careful shaving, disinfection with chlorhexidine, and surgical draping. The anterior part of the proximal right tibia was exposed through a short skin incision, and a small cortical bone hole was made next to the patellar tendon insertion using an injection needle. An intravenous catheter made of polytetrafluoroethylene (PTFE) (BD VenflonTM, Becton Dickinson Infusion Therapy, Helsingborg, Sweden) with the diameter of 1 mm was introduced into the medullary canal. The bacterial suspension was injected into the medullary cavity through the catheter. The group 1 rats received 0.05 mL solution of 3 × 10^8^ CFU/mL of *S. epidermidis*. Before the inoculation, a volume of 0.05 mL of 5% wt/vol. sodium morrhuate (Scleromate, Glenwood, Englewood, NJ, USA) was injected via the catheter. Sodium morrhuate is a sclerosing agent, composed of fatty acids and arachidonic acids, producing aseptic bone necrosis and increasing the probability of local bone infection. The animals of group 2 received equal 0.05 mL injections of *S. aureus* suspension (3 × 10^5^ CFU/mL) and aqueous sodium morrhuate. In group 3, no bacteria or aqueous sodium morrhuate were injected, but an equal amount of sterile saline was injected via the catheter into the medullary canal. Subsequently, the catheter was cut at the site of the cortical bone entry and the intramedullary portion of the catheter was left *in situ*. The wound was closed in layers. The anesthesia was reversed by subcutaneous injection of atipamezole hydrochloride (Antisedan 5 mg/mL, Orion Oyj, Espoo, Finland). During and after the surgery, the hypothermia of the animals was prevented using heating pads. Standard postoperative pain medication of buprenorphine (Temgesic® 0.3 mg/mL, PB Pharmaceuticals Limited, Slough, Berkshire, UK) was given subcutaneously for 3 days after the surgery. After the surgery, the functional activity of the animals was not restricted.

### Radiochemistry

DOTA-Siglec-9 peptide was purchased from Peptide Specialty Laboratories (Heidelberg, Germany). ^68^Ga was obtained from a ^68^Ge/^68^Ga generator (Eckert & Ziegler, Valencia, CA, USA) by elution with 0.1 M HCl. ^68^Ga eluate (0.5 mL, 290 to 350 MBq) was mixed with 2-[4-(2-hydroxyethyl)piperazin-1-yl]ethanesulfonic acid (HEPES; 120 mg) to give a pH of approximately 4.1. Next, 85 μg DOTA-Siglec-9 (35 nmol, dissolved in deionized water) was added, and the reaction mixture was heated at 100°C for 15 min. Radiochemical purity of ^68^Ga-DOTA-Siglec-9 was determined by reversed-phase high-performance liquid chromatography coupled with a radiodetector (radio-HPLC; Jupiter C18 column, 4.6 × 150 mm, 300 Å, 5 μm; Phenomenex, Torrance, CA, USA). The HPLC conditions were as follows: flow rate = 1 mL/min; *λ* = 215 nm; *A* = 0.1% trifluoroacetic acid (TFA)/water; *B* = 0.1% TFA/acetonitrile; and A/B gradient at 0 to 2 min 82/18, at 2 to 11 min from 82/18 to 40/60, at 11 to 14 min 40/60, at 14 to 15 min from 40/60 to 82/18, and at 15 to 20 min 82/18.

### PET/CT imaging

The imaging device was Inveon Multimodality PET/CT scanner (Siemens Medical Solutions, Knoxville, TN, USA). Two weeks after the surgery, rats were anesthetized with isoflurane and CT was performed for anatomical reference and attenuation correction. Subsequently, rats were intravenously injected with 19 ± 2.0 MBq of ^68^Ga-DOTA-Siglec-9 via the tail vein and a 30-min PET acquisition in a list mode was performed. PET data were reconstructed iteratively with the ordered-subsets expectation maximization 3D algorithm. A quantitative analysis was performed by a blinded observer (H.A.), and regions of interest (ROIs) were defined in the proximal and distal part of the operated, contralateral tibia and contralateral skeletal muscle using Inveon Research Workplace software (Siemens Medical Solutions, Malvern, PA, USA). The tracer accumulation was expressed as a standardized uptake value (SUV), i.e., [(average radioactivity within the ROI)/(injected radioactivity dose/rat body weight)]. The SUV_ratios_ between the operated tibia and the contralateral intact tibia and the operated tibia and contralateral muscle were calculated and used for intra- and inter-group comparisons.

**Figure 1:**
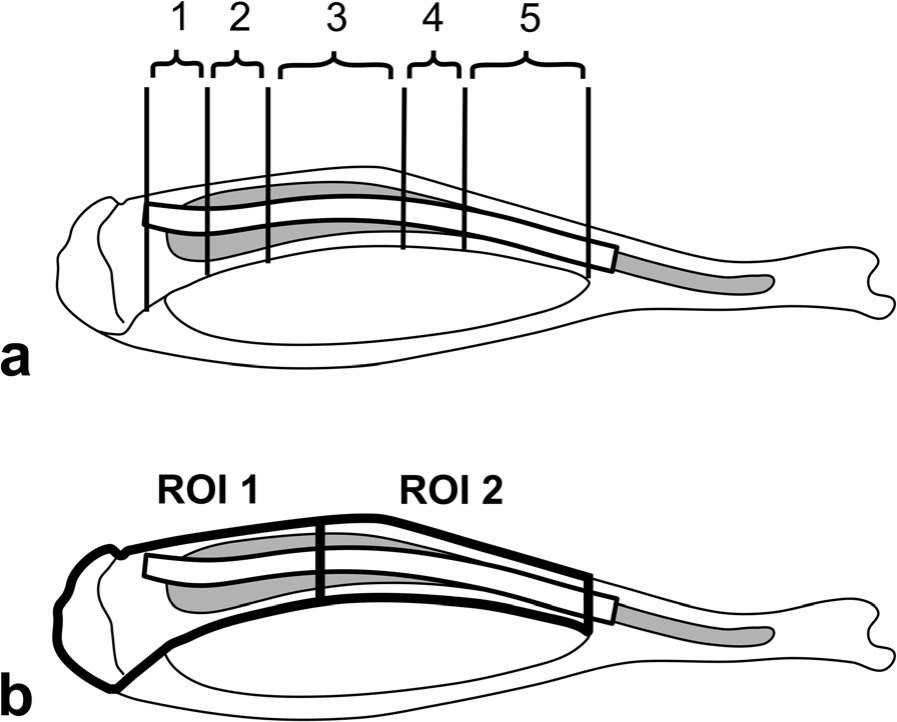
**Schematic illustration of the operated tibia. (a)** The bone was sliced into five sections for further characterization: (1) histological analysis (decalcified sections); (2) histological analysis (non-decalcified sections); (3) microbiological analysis; (4) *ex vivo* radioactivity measurement; (5) fluorescent microscopy of biofilm formation. **(b)** Quantification of the *in vivo* PET/CT data using two regions of interest (ROI).

Immediately after the PET imaging, the rats were sacrificed. The tibias with intramedullary catheters were retrieved and sliced into five segments using sterile techniques (Figure 1). The first two segments were taken for histology, the third segment was taken for microbiological analyses, the fourth segment was used for PET *ex vivo* radioactivity measurements, and the last fifth segment was prepared for fluorescence microscopy imaging of biofilm formation. Standard tissue samples (operated and contralateral tibia, contralateral muscle, blood, heart, kidney, liver, lung, plasma, and urine) were excised, weighed, and measured for total radioactivity using a gamma counter (1480 Wizard 3", PerkinElmer/Wallac, Turku, Finland). *Ex vivo* radioactivity measurements were corrected for the radionuclide decay to the time of injection. The radioactivity remaining in the tail was subtracted from the injected radioactivity. The tissue uptake of radioactivity was reported as a SUV and SUV_ratios_.

### Histology

The first bone segment of the retrieved tibias with the catheter *in situ* was used to prepare non-decalcified histological sections. The bone segment were fixed in 70% ethanol, dehydrated in a graded series of ethanol, cleared in xylene, and embedded in isobornylmehyacrylate (Technovit 1200 VLC, Kulzer, Germany) for sectioning and staining with van Gieson method. For the preparation of decalcified sections, the second bone segment was fixed in 10% formaldehyde, decalcified, embedded in paraffin, and stained with hematoxylin and eosin for the evaluation of inflammatory cell response. The stage of infection was semi-quantitatively graded using a scale system [23]: grade 0 = no infection, grade 1 = minimum evidence of infection, grade 2 = moderate evidence of infection, and grade 3 = severe infection. The histological grading was based on the consensus of three independent investigators.

### Microbiological analysis

The third bone segment of the retrieved tibia with the catheter *in situ* was used to prepare microbiological specimens. The bone was separated from the catheter. The bone specimens and the catheter specimens were placed into the separate tubes containing the fastidious anaerobe broth (FAB; LabM, Lancashire, UK). The bone specimens were kept in the freezer while the catheter specimens were incubated for 7 days under aerobic conditions, at 35°C. If visual bacterial growth was detected during the incubation period, then 50 μL of bacterial suspension was cultured on the blood agar plate and incubated overnight, at 35°C. Staphaurex latex agglutination test (Remel Europe Ltd, Dartford, Kent, UK) was performed for the identification of the isolated pathogens. If the agglutination test was negative, the analytical profile index (API ®/ID 32, BioMérieux SA, Marcy l'Etoile, France) was used for identification of staphylococcal colonies. If no visual bacterial growth was detected during the incubation time, the bone specimens were snap frozen with liquid nitrogen and pulverized with a mortar and a pestle. The bone chips were vortexed in saline for 5 min. The serial tenfold dilutions were taken. Subsequently, the samples were cultured on a blood agar plate and incubated for 48 h. If the culture results were negative, the polymerase chain reaction with universal 16S ribosomal DNA primers (16 s PCR) was performed, as previously described [24].

### Imaging with fluorescence microscope

The fifth segment of the retrieved tibia with the catheter *in situ* was taken for imaging with fluorescence microscope (Olympus BX 51; Olympus Optical Co Ltd, Hatagaya, Shibuya-ku, Tokyo, Japan). The bone content was separated from the catheter. The catheter was immersed in a 1-mL PBS, stained with live/dead staining (*Bac*Light kit™; Invitrogen, Barcelona, Spain) for 15 min without light access and then rinsed with PBS. The following staining conditions were used: 1.5 μL of SYTO® 9 (stock 3.34 mM dimethyl sulfoxide, DMSO) and 1.5 μL propidium iodide (stock 20 mM DMSO) in 1 mL PBS. During the imaging with fluorescence microscope, SYTO® 9 green fluorescence marked the living microorganisms with intact membrane and propidium iodide red fluorescence marked the dead bacteria with damaged membrane. After the staining procedure, the cells were imaged.

### Statistical analyses

Normal distribution of the data was verified using Kolmogorov-Smirnov test. A paired *t* test was used in the intra-animal comparison of the tracer uptake between the operated and contralateral bones. One-way ANOVA with Tukey's post hoc tests were used in the inter-group comparisons of the PET data. Non-parametric Kruskal-Wallis test with Mann-Whitney post hoc tests were used in the inter-group comparison of the histological data. Non-parametric Spearman rank-order correlation analysis (two-tailed) was used to examine associations between the PET and histological data. Statistical analyses were done using IBM SPSS statistical software (version 19, SPSS Inc, Chicago, IL, USA).

## Results

### Measurements for biofilm production

Prior to the *in vivo* experiments, the bacterial strains were tested for their *in vitro* capability to form biofilms. After 24 h of incubation, both strains were able to form the biofilm. Moreover, there were no significant differences in biofilm mass and, therefore, in the capacity of biofilm production between the *S. epidermidis* and *S. aureus* strains.

### Radiochemistry

According to radio-HPLC, the radiochemical purity of ^68^Ga-DOTA-Siglec-9 was ≥95% throughout the study. Under the conditions described above, the retention time of ^68^Ga-DOTA-Siglec-9 was 9.8 ± 0.04 min.

### PET imaging

#### Inflammatory response to sterile catheters

The ^68^Ga-DOTA-Siglec-9 PET/CT imaging was able to detect the inflammation/bone healing process induced by implantation of the indwelling catheters. Group 3 (*n* = 10) showed an increased local uptake of the ^68^Ga-DOTA-Siglec-9 in the operated tibias compared with the intact contralateral bones. The differences were significant both in the proximal (SUV_ratio_ +29.5%, *p* < 0.001) and distal (SUV_ratio_ +23.7%, *p* < 0.001) tibia (ROI1 and ROI2, respectively).

**Figure 2:**
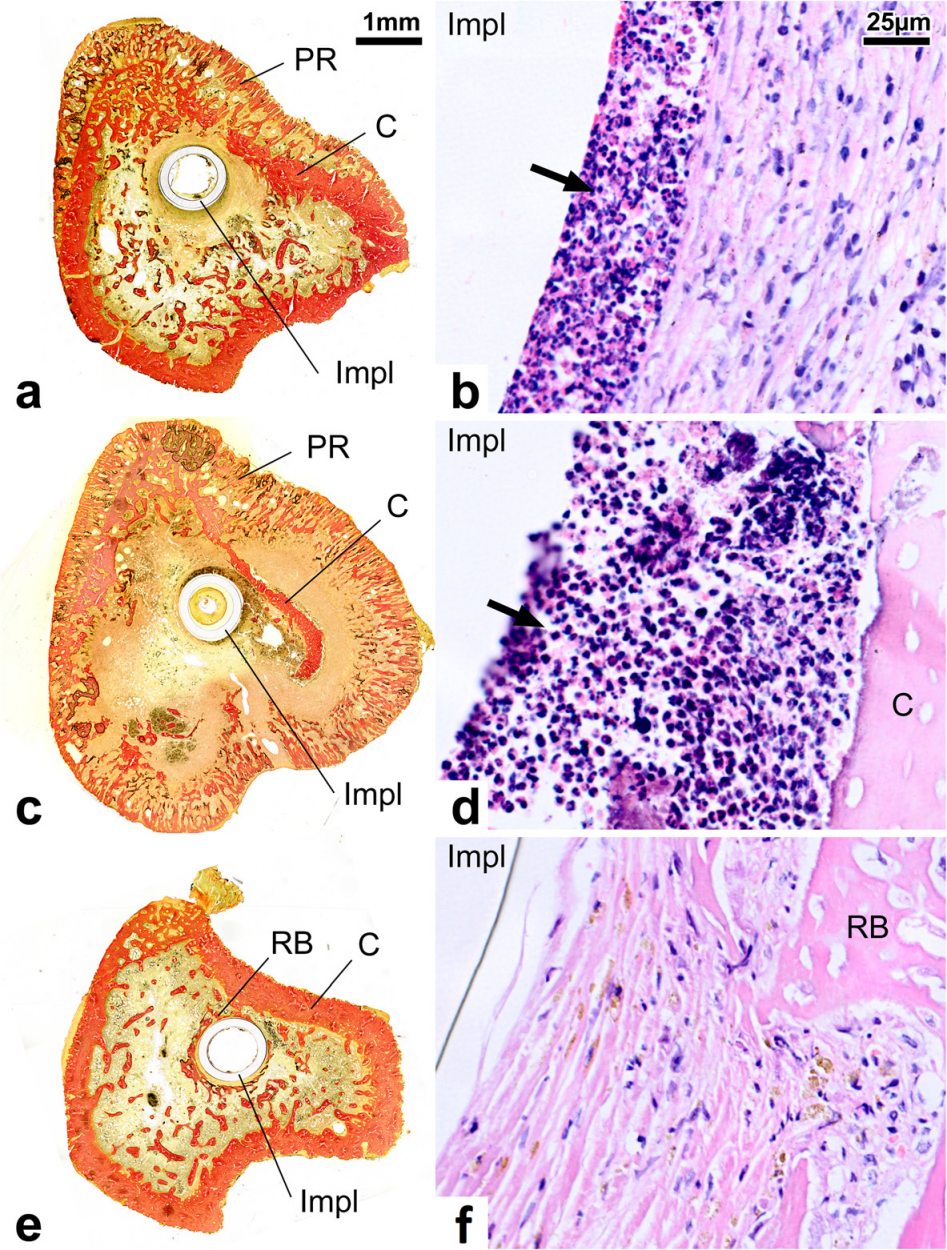
**Histological-analysis. (a)***Staphylococcus epidermidis* group, van Gieson stain. Inflammatory response is expressed as a sunburst type periosteal reaction (PR) and partial resorption of the cortex (C). Implant is denoted as ‘Impl’. **(b)***S. epidermidis* group, hematoxylin and eosin stain. Increased number of polymorphonuclear leukocytes were observed in the medullary canal in the proximity of the implant (arrow). This layer is surrounded by granulation tissue; **(c)***Staphylococcus aureus* group, van Gieson stain; Inflammatory response is expressed as a circumferential sunburst type periosteal reaction (PR) and an almost complete resorption of the cortex (C). **(d)***S. aureus* group, hematoxylin and eosin stain. Polymorphonuclear leukocytes are seen in the proximity of the implant (arrow). **(e)** Sterile catheter implant group, van Gieson stain; Periosteal and cortical reactions are absent. Reactive bone formation (RB) is seen around the implant. **(f)** Sterile catheter implant group, hematoxylin and eosin stain. The implant is surrounded by fibrous capsule and reactive bone (RB).

**Figure 3:**
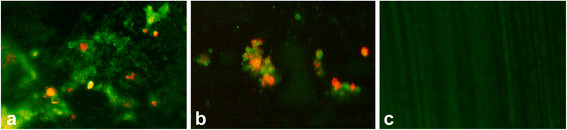
**Fluorescence microscope images of catheters.** The catheter surfaces stained with BacLite Kit. Biofilm clusters composed of aggregates of viable coccoid bacterial cells, which were stained with SYTO® 9 (green color) and dead bacteria stained with PI (orange-red). **(a)***S. epidermidis*; **(b)***S. aureus*; **(c)** sterile catheter.

Histologically, there was a low inflammatory reaction and reactive new bone formation around the catheters (Figure 2e,f). No bacteria could be cultured from the retrieved catheters and bone specimens. Except for one sample with probable contamination, the catheters showed no bacterial biofilm in fluorescent microscopy (Figure 3c).

#### Inflammatory response to *S. aureus* peri-implant infection

The ^68^Ga-DOTA-Siglec-9 PET/CT imaging detected inflammatory response to the implant-related infection caused by *S. aureus* in the proximal (SUV_ratio_ +41.7%) and distal (SUV_ratio_ +30.9%) tibia (ROI1 and ROI2, respectively). However, due to the limited group size (*n* = 7) following failed tracer injections in three animals and high scatter in the data, the differences were not statistically significant. Subsequently, in the inter-group comparison of the PET data, the difference between *S. aureus* group and group 3 without bacterial inoculation was not statistically significant, even when the animals with the negative microbiological results were excluded from the analysis.

The histological appearance of the infection sites ranged from low to severe reaction; in general, the infection was severe (median grade 3, range 1 to 3). In the most extreme cases, the infection was manifested in significant periosteal reaction, extensive destruction of the cortex, and increased number of the polymorphonuclear leukocytes in the medullary canal (Figure 2c,d). In the inter-group comparison of the histological data, there were statistically significant differences between *S. aureus* group and group 3 without bacterial inoculation (*p* < 0.001).

There was a positive bacterial growth for the inoculated *S. aureus* strain in 70% and 60% of the catheter and bone specimens, respectively. The animals with negative cultures also had negative PCRs. In fluorescent microscopy imaging, the presence of a biofilm on the surface of the retrieved catheters was observed 70% of the specimens (Figure 3b).

#### Inflammatory response to *S. epidermidis* peri-implant infections

**Figure 4:**
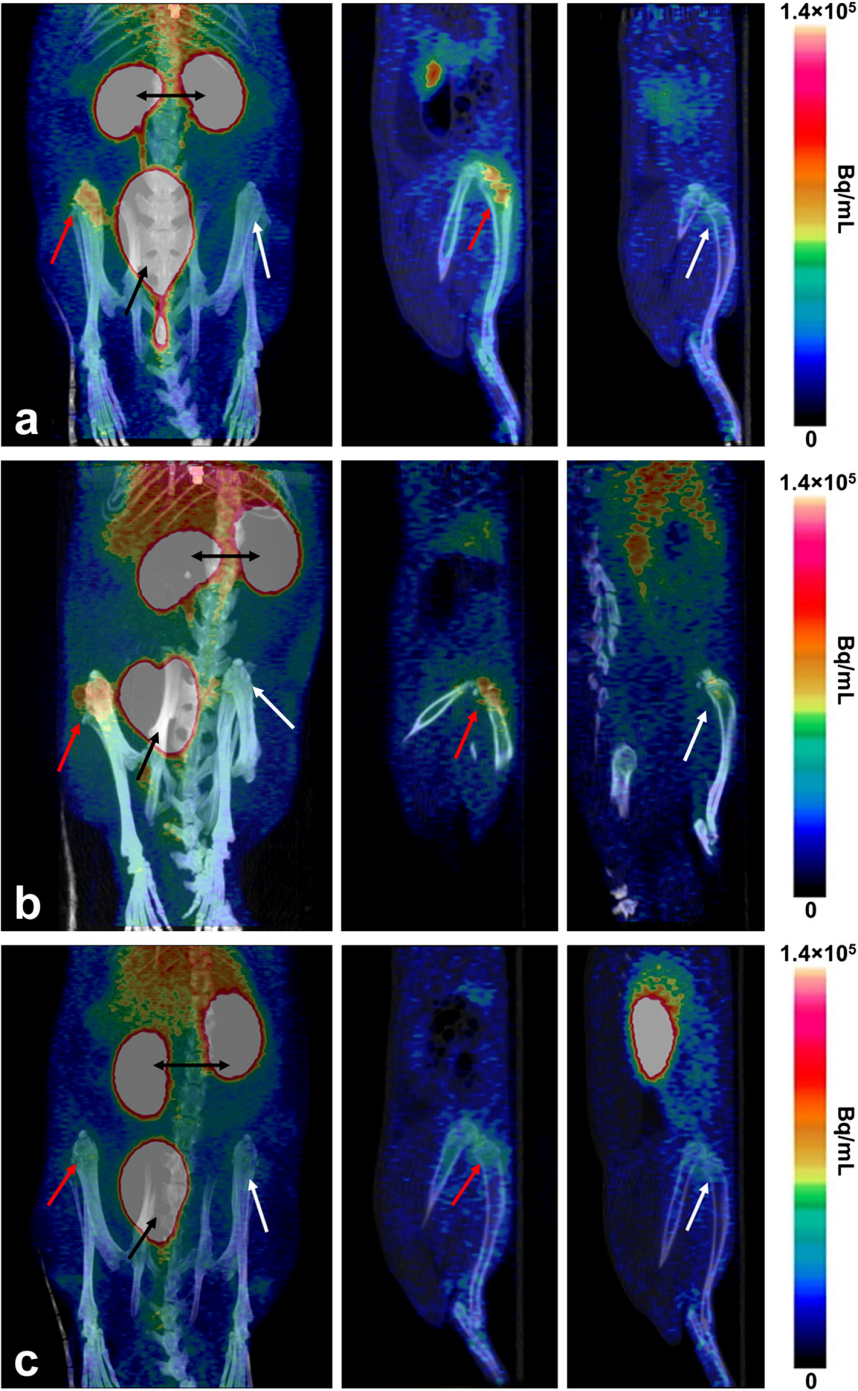
**Representative sagittal and coronal PET/CT images.** Representative sagittal and coronal PET/CT images with ^68^Ga-DOTA-Siglec-9 peptide of the rats with **(a)** catheter-related *S. epidermidis* infection of the right tibia, **(b)** catheter-related *S. aureus* infection of the right tibia, or **(c)** catheter implantation in the right tibia without bacterial inoculation. High focal uptake of radioactivity in the infected right tibia is observed (red arrows) compared with the contralateral intact left tibia (white arrows). Excess of radioactivity is excreted through the kidneys (two headed black arrow) to the urinary bladder (black arrow).

**Figure 5:**
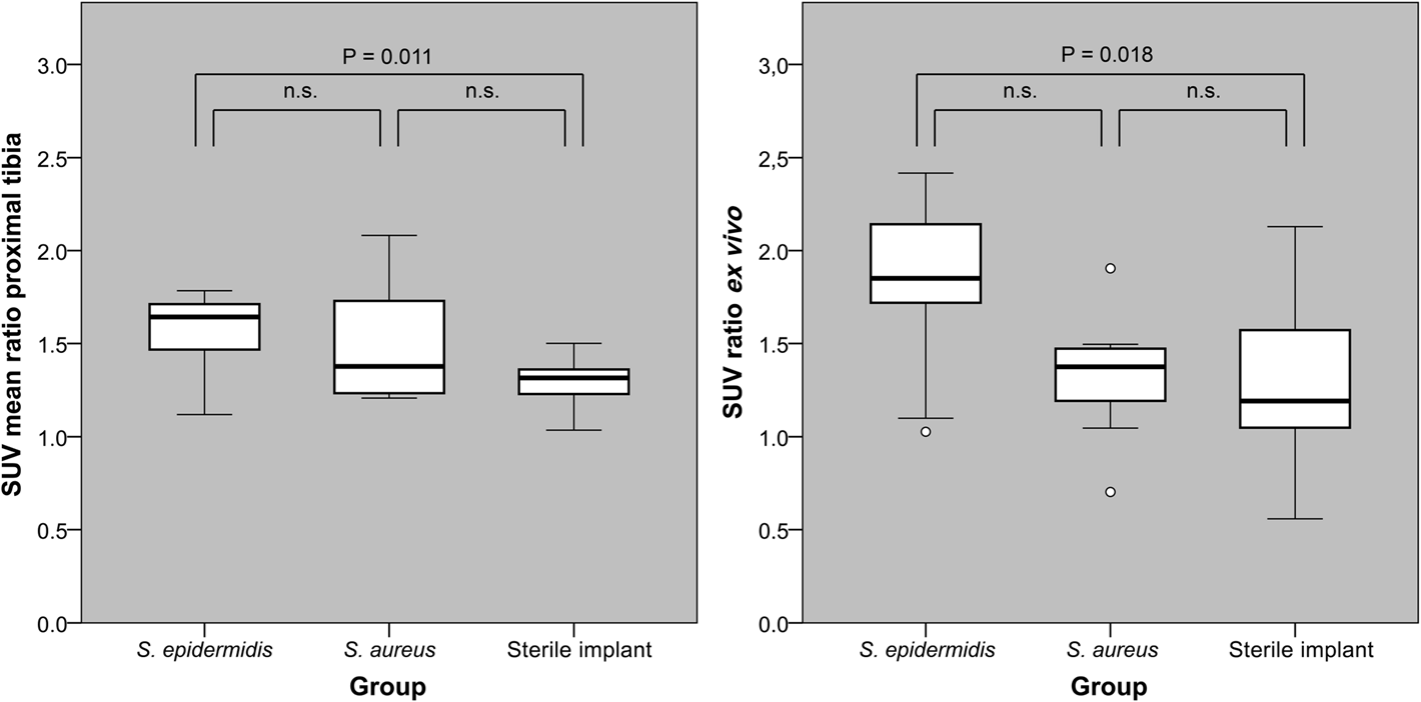
**Comparison of the uptake of**^**68**^**Ga-DOTA-Siglec-9 in the three groups of animals.** The three groups are as follows: *S. epidermidis* infection, *S. aureus* infection, and sterile catheter implant. The uptake is shown as the intra-animal SUV_ratio_ values of the proximal tibias measured *in vivo* (ROI1 in Figure 1) and *ex vivo* (section 4 in Figure 1). Box plots of are showing median, first and third quartiles, minimum and maximum values, and outliers. Comparison between the groups performed with ANOVA with Tukey's post hoc test.

The ^68^Ga-DOTA-Siglec-9 PET/CT imaging detected inflammatory response to the implant-related infection caused by *S. epidermidis*. The animals with peri-implant *S. epidermidis* inoculations (*n* = 10) showed the highest local uptake of the ^68^Ga-DOTA-Siglec-9. Compared with the intact contralateral bones, the difference in the tracer uptake was significant both in the proximal (SUV_ratio_ +58.1%, *p* = 0.009) and distal (SUV_ratio_ +48.2%, *p* = 0.013) tibia (ROI1 and ROI2, respectively). ^68^Ga-radioactivity was accumulated especially in the proximal part of the tibia (Figure 4a). In the inter-group comparison, the uptake of ^68^Ga-DOTA-Siglec-9 in the proximal tibia was significantly higher in the *S. epidermidis* group than in group 3 without bacterial inoculation (*p* = 0.020) (Figure 5). The difference in the tracer uptake between the *S. epidermidis* group and group 3 was significant (*p* = 0.005) also in the distal part of the tibia (ROI2). In addition, when the PET data of *S. epidermidis* group and *S. aureus* group were pooled together, statistical comparison with group 3 without bacterial inoculation revealed significant differences in the tracer uptake (*p* = 0.030 in the proximal part of the tibia and *p* = 0.037 in the distal parts of the tibia).

Histologically, in *S. epidermidis* group, there were signs of severe infection (median grade 3, range 1 to 3), including circumferential sunburst-type periosteal reaction, moderate subperiosteal, endosteal, and intracortical resorption of the cortex, and enlarged Haversian canals filled with granulation tissue and fragmented polymorphonuclear leukocytes with occasional microabscesses (Figure 2a,b). However, the extensive destruction of the cortex characteristic to the *S. aureus* infection (Figure 2c) was not observed in *S. epidermidis* group (Figure 2a). In the inter-group comparison of the histological data, there were statistically significant differences between *S. epidermidis* group and group 3 without bacterial inoculation (*p* < 0.001). The difference between *S. epidermidis* group and *S. aureus* group was not statistically significant.

Spearman rank-order correlation revealed statistically significant associations between SUV_ratio_ and histological data for proximal (*R*_s_ = 0.565, *p* = 0.003) and for distal (*R*_s_ = 0.629, *p* = 0.003) parts of the tibia when the data was analyzed *en bloc*. However, if the data were split into the three groups (*S. epidermidis* group, *S. aureus* group, and group with catheter implantation without bacterial inoculation), the statistically significant associations were no longer present.

Fluorescent microscopy demonstrated the presence of a biofilm on the surface of the retrieved catheters in 82% of the animals in the *S. epidermidis* group (Figure 3a). In microbiological examination, specimens from all animals, except one, showed positive bacterial growth in catheter and bone samples. The API test verified the presence of bacteria.

## Discussion

*S. epidermidis*, an innocuous commensal habitant of the human skin and mucous membranes, has emerged as a frequent cause of nosocomial infections [25]. *S. epidermidis* is the most common origin of infections of indwelling medical devices [25], in particular, periprosthetic joint infections and intravascular catheter-related bloodstream infections [1],[2]. These infections pose high challenges for microbiologic studies and diagnostic imaging. Indeed, our recent animal study demonstrated that *S. epidermidis* bone infections were characterized by low ^18^F-FDG uptake in PET/CT imaging, reflecting the limited inflammatory host response to the pathogen [16]. The rationale of the current experiment was to explore a novel approach to detect the inflammatory response to *S. epidermidis* peri-implant infections by means of PET imaging of leukocyte trafficking using VAP-1 as the target molecule. The results indicate that ^68^Ga-DOTA-Siglec-9 PET was able to detect the inflammatory response to *S. epidermidis* peri-implant infections.

The imaging of leukocyte trafficking using VAP-1 as a target molecule is a novel approach. VAP-1 is an inflammation inducible endothelial cell molecule mediating leukocyte interactions with the lining of blood vessels [17],[19]. It contributes to several steps in the extravasation cascade and controls trafficking of lymphocytes, granulocytes, and monocytes to sites of inflammation. VAP-1 is practically absent from the endothelial surface of normal tissues [17],[19]. Previously, we have reported the *in vivo* stability, tissue distribution, and bio-kinetics of the VAP-1-targeting peptides for PET imaging of inflammation in animal models [26]-[29]. The ability to image inflammation was shown in a rat bone healing model [26]. Using a phage display approach, we have discovered that Siglec-9 is a granulocyte ligand for VAP-1 and a ^68^Ga-labeled Siglec-9 motif peptide specifically detects VAP-1 in vasculature at sites of inflammation and cancer by PET [29]. Although granulocytes can bind to endothelium via a VAP-1-dependent manner, the counter-receptor(s) on this leukocyte population were not known before.

An animal model of acute peri-implant osteomyelitis was modified for this study. The model relies on the use of a sclerosing agent, sodium morrhuate, to promote the development of infection. The use of sodium morrhuate for the promotion of osteomyelitis has been criticized [30],[31]. For example, in a previous report, histological examination, performed 2 weeks postoperatively, detected a slight increase in the periosteal bone formation in rat tibias treated with sodium morrhuate [32]. The bone repair process, which follows the aseptic bone necrosis caused by the sclerosing agent, could be misinterpreted in diagnostic imaging, especially at the early stages after inoculation [30]. Despite this drawback, the animal model with sodium morrhuate pretreatment is considered robust and reproducible [31]. Therefore, it has been frequently applied in the studies involving diagnostic imaging without an animal group dedicated to the assessment of the effect of sodium morrhuate alone [16],[27],[32]-[34]. The virulence of slime-producing *S. epidermidis* strains can be so low that even the use of aqueous sodium morrhuate does not guarantee the induction of bone infection. In our previous experiment [16], using a rabbit model with a small block of bone cement as the foreign body and use of adjunct sodium morrhuate, we observed that a standard slime-producing laboratory *S. epidermidis* strain (ATCC 35983) induced only occasionally culture-positive bone infections, while the clinically retrieved *S. epidermidis* strain (T-54580) produced infection in a reliable manner. In this study, we cannot exclude the possibility that sodium morrhuate caused inflammation on its own and acted as a confounding factor. However, the lack of statistically significant differences in the tracer uptake between *S. aureus* group with sodium morrhuate pretreatment and the sterile implant group without sodium morrhuate pretreatment suggests that the effect of the sclerosing agent is marginal.

Silicone catheter is one of the commonly used foreign bodies in animal models of osteomyelitis [35]-[37]. Lambe and colleagues [37] used this model to induce osteomyelitis in the rabbit tibia with *S. epidermidis*. To our knowledge, this is the first study to report the use of catheters in rat models of osteomyelitis. The main benefit was the closed direct injection of the inoculum into the close space of the medullary canal. The use of an injection needle for the creation of a hole in the metaphysis eliminates the need to seal the aperture [31].

In *S. aureus* group, 10^4^ CFU inoculum (3 × 10^5^ CFU/mL, 0.05 mL) was above the minimum level of 10^3^ CFU reported to produce implant-related infections [38]. In our previous experiment, the same inocula induced infection in the rabbit tibia with a foreign body (a small block of bone cement) [16]. In rats, 10^4^ CFU inoculum was reported to produce implant-related infections [39]. Based on the microbiological and histological findings of this study, the infection was detected in 60% to 70% of the animals. The inconstant induction of *S. aureus* osteomyelitis may be related to the selected dose. In a previous experiment of the rat tibia, we applied a higher dose (10^7^ CFU), which caused severe osteomyelitis in all cases [34].

The inoculum 10^7^ CFU (3 × 10^8^ CFU/mL, 0.05 mL) of the slime-producing clinical isolate of *S. epidermidis* was used in this study. In the rabbit models of osteomyelitis with *S. epidermidis*, bacterial inocula have been in the range of 10^4^ to 10^8^ CFU [16],[37],[40]. Rat models of *S. epidermidis* osteomyelitis are less common [41]. In an implant-related osteomyelitis model, 10^5^ CFU inoculum was reported for *S. epidermidis*[41]. In our study, a 10^7^ CFU inoculum was selected based on the results of our pilot study (unpublished data), which showed a consistent foreign-body-associated (bone cement block) osteomyelitis in the rat tibia in response to the adjunct inoculum of 10^8^ CFU of *S. epidermidis*. The dose was lowered in the current experiment because the goal was to create a low-grade biofilm-related infection commonly encountered in the clinical settings. Probably reflecting the efficient closed administration of the bacterial inoculum via the catheter, even the lowered dose led to the unexpectedly severe osteomyelitis. Thus, this experiment did not simulate the PET/CT imaging of typical clinical low-grade *S. epidermidis* infections and we cannot be sure that the applied imaging technique detects low-grade *S. epidermidis* infections.

WBC scans have emerged as the leading technique for imaging of periprosthetic joint infections [9],[10]. Optimization of the imaging and interpretation protocols of WBC scans allows an improved differentiation of sterile inflammation from infection-related accumulation of leukocytes [42],[43]. The specificity of ^18^F-FDG for differentiation between inflammation and infection is limited [44] and in the field of orthopedic surgery, the main disadvantage of the current techniques of ^18^F-FDG-PET/CT imaging relates to the inability to differentiate bacterial infections and aseptic inflammatory processes caused by mechanical loosening of joint prostheses [12],[45]. This will also be the challenge for the development of ^68^Ga-DOTA-Siglec-9 PET/CT techniques to become a useful clinical tool.

## Conclusions

Based on this exploratory study, ^68^Ga-DOTA-Siglec-9 PET is able to detect inflammatory tissue response induced by catheter-related *S. epidermidis* infection. One of the next preclinical steps will be the modification of the current animal model to achieve a model of low-grade *S. epidermidis* peri-implant infection and to compare the uptake of ^68^Ga-DOTA-Siglec-9 in a model of inflammation simulating aseptic loosening of bone implants.

## Competing interest

The authors declare that they have no competing interests.

## Authors' contribution

HA carried out the PET/CT imaging and participated in the data analysis and helped to draft the manuscript. JK contributed in the design of the microbiological analyses and carried out imaging with a fluorescence microscope and participated in the data analysis and helped to draft the manuscript. EE and AJH participated in the design of the microbiological analyses. NM participated in the experimental surgery and performed the statistical analysis. MS performed the histopathological analysis. TS participated in the PET imaging. SJ and AR participated in the design of the study and drafting the manuscript. HTA conceived the study and participated in its design, performed the experimental surgery, and drafted the manuscript. All authors read and approved the final manuscript.
